# Enhancing Plum Wine Safety and Aroma Using Pulsed Electric Field Pretreatment

**DOI:** 10.3390/molecules30224393

**Published:** 2025-11-13

**Authors:** Jian Li, Hua-Xi Huang, Dan-Li Tang, Xin-An Zeng, Lang-Hong Wang, Man-Sheng Wang

**Affiliations:** 1 Guangdong Provincial Key Laboratory of Intelligent Food Manufacturing, School of Food Science and Engineering, Foshan University, Foshan 528225, China; li_jian@fosu.edu.cn (J.L.); 14767786114@163.com (H.-X.H.); xazeng@scut.edu.cn (X.-A.Z.); 2 School of Food Science and Engineering, South China University of Technology, Guangzhou 510641, China; 17860730737@163.com; 3 Institute of Bast Fiber Crops, Chinese Academy of Agricultural Sciences, Changsha 410205, China

**Keywords:** green plum wine, pulsed electric field, bitter amygdalin, cyanide, aroma compounds

## Abstract

Traditional soaking plum wine production is time-consuming and often results in high levels of bitter amygdalin and toxic cyanide, posing health risks. In this study, response surface methodology (RSM) with a Box–Behnken design was employed to optimize pulsed electric field (PEF) parameters, developing a novel process integrating kernel detoxification and PEF pretreatment to mitigate these hazards, enhance the characteristic aroma (benzaldehyde), and shorten the maceration cycle. The experimental results showed that the contents of bitter amygdalin and cyanide in plum kernels after detoxification and PEF pretreatment were reduced by 62.34% and 59.62%, respectively, compared with the control group, and the contents of both were further reduced with the addition of plum flesh for further soaking in the new process. In addition, the PEF pretreatment also increased the amount of benzaldehyde extracted by 4.63% compared to the control group and resulted in a 10.53% reduction in equilibration time. Moreover, compared to the previous whole-fruit maceration process, the new process resulted in a 37.5% reduction in the final plum wine production cycle. This study provides a practical solution for improving the safety and efficiency of plum wine production and supports the industrial application of PEF technology.

## 1. Introduction

Green plums (*Prunus mume*), commonly known as “sour plums” and “fruit plums”, are a common raw material for Chinese plum wine, which is believed to eliminate fatigue; regulate the body; and act as a detoxifying, laxative, appetizing, and anti-allergic agent, among other benefits [1,2]. Currently, most distilleries use the traditional whole-fruit infusion method to make plum wine. While this method is relatively simple and efficient, its production cycle usually takes more than three months, or even longer [3]. Most importantly, the production process of soaking plum wine is generally characterized by high levels of bitter amygdalin and cyanide, which pose a risk to the health of drinkers [4]. Usually, bitter amygdalin exists in the seeds of plants of the Rosaceae family, and is especially abundant in the kernel of plums, up to 2.08%. Amygdalin is hydrolyzed to prunasin by β-glucosidase amygdalin hydrolase, releasing glucose (Glc) as a byproduct. Prunasin is then further hydrolyzed by prunasin hydrolase (another β-glucosidase) to yield mandelonitrile and glucose. Mandelonitrile is subsequently converted into benzaldehyde and hydrogen cyanide (HCN) by mandelonitrile lyase, and HCN ultimately transforms into hydrocyanic acid, which imparts a strong bitter taste to wine. The solubility of benzaldehyde is influenced by its glycosidic structure, while its low solubility is attributed to its aromatic and hydrophobic nature, which affects its extraction efficiency [5,6]. Likewise, cyanide also exists mainly in the kernels, with a very small amount in the flesh of the fruit, and the human body experiences different degrees of toxicity after ingesting cyanide, which may lead to death in serious cases [7]. Alcohol dilution is commonly used to reduce the concentration of amygdalin and cyanide. Unfortunately, this method negatively affects the flavor and texture of plum wine. In addition, researchers have attempted to eliminate amygdalin and cyanide using enzymatic methods, but the high cost of amygdalinase has limited its widespread use in industry [8,9]. Therefore, finding a more cost-effective way to remove amygdalin and cyanide from plum wine that maintains its flavor and effectively shortens the maceration period remains an industry challenge.

The pulsed electric field (PEF), as a novel non-thermal processing technology for food processing, has been widely studied. The main working principle is to apply a high-voltage transient pulsed electric field (1~80 kV/cm) to the material placed between the two electrodes, which causes the cell membrane of the material to undergo an electroporation effect. This unique electric field effect can change the texture of the material, promote the solvents in the external environment to enter the material, and dissolve the active cellular ingredient in the material [10,11]. Therefore, PEF technology has the ability to assist in shortening the production cycle and facilitating the extraction of functional ingredients [12]. It has been reported that PEF can significantly increase the mutual solubility of extraction solvents and flavor components as well as the ability of the components to pass through the cell wall, thus improving the extraction efficiency of the flavor components. Piergiorgio et al. [13] indicated that a total specific energy of 22 kJ/kg PEF processing enabled more intense extraction of varietal aroma precursors without provoking excessive color evolution and extraction of phenolic compounds, apparently increasing the stability of wine towards oxidation. As one of the characteristic aromas of plums, benzaldehyde gives plum wine its typical “plummy” flavor [14], but the distribution of benzaldehyde in plums remains poorly understood. A previous study on Red-Fleshed Sweet Cherries (*Prunus avium var. Stella*) showed that treatment with a pulsed electric field of 2.5 kV/cm significantly increases the benzaldehyde concentration due to the enhanced electrodialysis induced by the high electric field intensity, as well as the stimulation of enzymatic metabolic activity within plant cells [15]. However, whether the assisted immersion extraction of PEF would increase the content of benzaldehyde in plum wine and thus make the aroma of the wine more intense deserves further investigation. Otherwise, previous studies pointed out that effective detoxification of bitter amygdalin and cyanide could be achieved by soaking in distilled water [16], with bitter amygdalin and cyanide being readily soluble in water, and benzaldehyde being slightly soluble in water. Thus, we try to utilize the difference in water solubility of the three to remove the harmful ingredients in green plums.

Based on the above considerations, the main objectives of this study were to select the raw materials for producing soaked plum wine by comparing the aroma components of green plum pit, yellow fruit flesh, and green fruit flesh, and to optimize the maceration method to reduce the content of bitter amygdalin and cyanide in soaking plum wine. The parameters of PEF-assisted extraction of benzaldehyde were systematically optimized employing RSM (Box–Behnken design) and to develop an innovative process to increase the content of benzaldehyde in plum wine while speeding up the maceration production cycle. This study aims to provide a reference for improving and optimizing the production process of green plum wine.

## 2. Results

### 2.1. Aroma Components of Plum Kernel and Flesh

**Figure 1:**
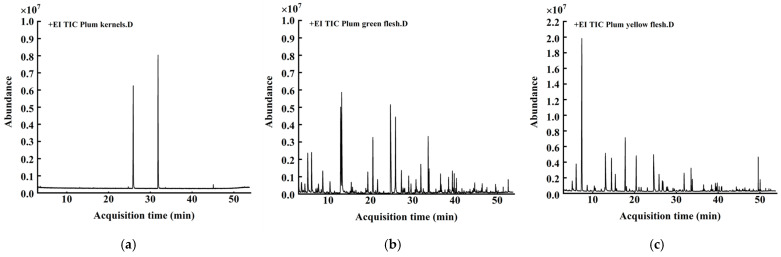
Total ion chromatogram of aroma components in kernel and flesh of plum: (**a**) plum kernel; (**b**) green flesh; (**c**) yellow flesh.

Aroma is one of the most important characteristics of fruit wines, which is the main determinant of consumer preference and plays a crucial role in determining the organoleptic quality and style of fruit wines. Different fruit wines present distinctive aroma profiles, which are mainly determined by the type and content of aroma compounds. As one of the characteristic aromas of plum, benzaldehyde gives plum wine a typical “plum aroma” flavor [17]. Thus, in this part, we measured and compared the aroma compositions of plum kernel and plum green flesh and yellow flesh (where yellow is the color of a more mature plum), aiming to investigate the distribution of benzaldehyde in plum and the difference in aroma compositions between green and yellow flesh so as to provide a basis for raw material selection and design for a new method of soaking plum wine. Figure 1 shows the total ion flow pattern of plum kernel, green flesh, and yellow flesh samples determined using headspace solid-phase microextraction with GC. It can be seen that there are certain differences in the types and richness of aroma components among different raw materials, and the relative contents of benzaldehyde in the kernel and green and yellow flesh are shown in Table 1, Table 2 and Table 3, indicating that benzaldehyde is almost completely distributed in plum kernels.

Otherwise, 36 aroma components were detected in green flesh, and the key aroma components were ethyl butyrate, 1-octen-3-ol, (E)-2-octenal, (E)-2-heptenal, 1-hepten-3-one, hexanal, β-damascenone, and D-limonene, with the OVA values being 106.20, 35.30, 18.23, 10.76, 7.13, 6.24, 2.30, and 1.24, respectively (Table 2). Comparatively, a total of 40 aroma components were detected in yellow flesh, with the key aroma components including ethyl butyrate, hexyl acetate, butyl acetate, foliate acetate, β-damascenone, R-γ-Decalactone, and γ-Dodecanolactone. The OAV was 347.00, 307.55, 31.31, 13.35, 4.50, 1.58, and 1.29, respectively (Table 3), which shows that the OAV of the key aroma components in the yellow flesh is greater, indicating the aroma characteristics are more prominent in it. Among them, ethyl butyrate was the primary aromatic substance in the plum, which had a strong sweet fruity aroma [19], and its content in yellow flesh was 3.27 times that in green flesh, and β-damascenone in yellow flesh was 1.96 times that in green flesh, which is another key aroma component highly similar to that of roses [20]. Moreover, the key aroma-presenting substances in green flesh are mostly aldehydes, including (E)-2-octenal, (E)-2-heptenal, hexanal, etc., which are not prominent in aroma. In contrast, the key aroma-forming substances in yellow flesh are mostly esters, such as ethyl butyrate, hexyl acetate, etc., presenting a pleasant floral and fruity flavor, which means that the ripe flesh is richer in flavor substances. Similar results were shown in a study conducted by Song et al. [21], who found that the most volatile organic compounds (VOCs) were highly accumulated in mid-ripe and ripe feijoa fruits, with the percentage of esters and terpenoids being higher in ripe feijoa, leading to a strong fruity and flower fragrance. According to research by Hadi et al. [22], this phenomenon can be explained by the biosynthetic pathway in which fatty acids act as primary precursors for aroma volatiles in most fruits. Their derivatives, including straight-chain alcohols, aldehydes, ketones, acids, esters, and lactones, are largely formed via α-oxidation, β-oxidation, and the lipoxygenase (LOX) pathway. These compounds collectively define the fruit’s aroma profile and sensory character. During ripening, the activation of the LOX pathway in intact plant tissue provides an alternative to β-oxidation, thereby significantly increasing the concentration of aroma compounds.

Based on the results of the aroma components, the new process for soaking green plum wine was decided to use more mature yellow flesh and to separate the flesh from the kernel to improve soaking efficiency.

### 2.2. Detoxification Pretreatment of Plum Kernels

Previous studies have shown that plum kernels contain mainly bitter amygdalin and cyanide [23,24], but in this study, we also found that benzaldehyde, an aromatic compound specific to plums, is also almost completely concentrated in the kernels. Inspired by previous studies, we aimed to directly remove bitter amygdalin and cyanide from plum kernels via immersion in distilled water, taking advantage of the difference in solubility of the three substances in water—bitter amygdalin has a water solubility of 83.0 g/L at 25 °C, cyanide is highly soluble in water (for example, HCN can miscible with water in all proportions at standard room temperature and pressure), and benzaldehyde has limited solubility in water (3.3 g/L at 25 °C) [25]—to retain as much benzaldehyde as possible.

**Figure 2:**
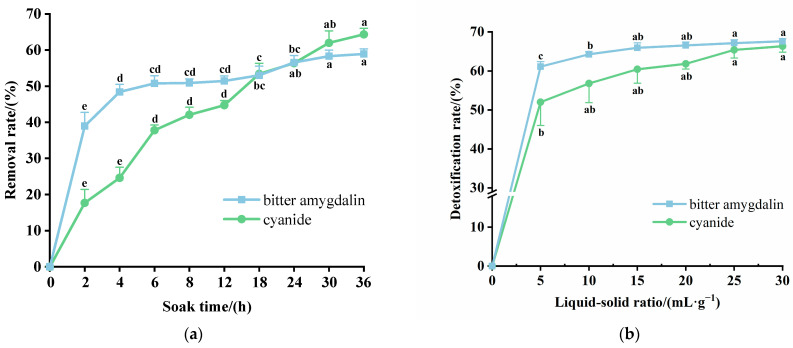
Pretreatment for whole-kernel detoxification of green plum: (**a**) soaking time; (**b**) liquid–solid ratio. Different letters indicate significant differences (*p* < 0.05).

As shown in Figure 2a, the effect of soaking time on the removal of bitter amygdalin and cyanide from plum kernels was first investigated. Between 0 and 6 h, the longer the soaking time, the higher the removal rate, especially for amygdalin. After 24 h of soaking, the removal of bitter amygdalin reached 56.56%, but the removal of cyanide continued to increase, and then reached 61.97% after 30 h of soaking. Considering the overall effect of soaking time on the removal of bitter amygdalin and cyanide from plum seeds, 30 h was the optimum soaking time. As a result, bitter amygdalin removal reached 58.32%, and cyanide removal reached 61.97%. Figure 2b illustrates the effect of varying the proportion of distilled water soaking on the removal of bitter amygdalin and cyanide from kernels. Both the removal of bitter amygdalin and cyanide continued to increase with an increase in the proportion of distilled water. The removal of cyanide reached an equilibrium of 56.80% when the liquid/solid ratio reached 10, and bitter amygdalin comprised 64.26%. When the liquid/solid ratio was 15, the removal of bitter amygdalin and cyanide reached 65.93% and 60.44%, respectively. The values did not increase significantly when increasing the proportion of distilled water. Thus, the distilled water immersion at a ratio of 15:1 (mL:g) resulted in the best removal of bitter amygdalin and cyanide from plum kernels, which suggested that distilled water immersion is effective in removing toxic substances from plum seeds, which is similar to the results of previous studies, which found that the degradation products of amygdalin were dissolved in large quantities in the immersion water. Silem et al. indicated that amygdalin was efficiently degraded by endogenous β-glycosidases released from plant material during immersion, leading to the dissolution of approximately 70% of amygdalin into the soaking water [26,27]. Therefore, our preferred parameters for the detoxification process were soaking in distilled water for 30 h and a liquid–solid ratio of 15:1 (mL:g).

### 2.3. PEF-Assisted Extraction of Benzaldehyde

After selecting the optimal distilled water soaking time and ratio for the detoxification pretreatment of plum kernels, the characteristic aroma component benzaldehyde was extracted from the kernel using PEF assisted by food-grade alcohol soaking to investigate whether the synergistic extraction with PEF could increase the amount of benzaldehyde content. The effects of four single factors, i.e., electric field strength, pulse number, pulse frequency, and pulse width, on the benzaldehyde content in the soaking solution were investigated, and the response surface optimization test was carried out to optimize the final selection of the parameters of the PEF treatment.

**Figure 3:**
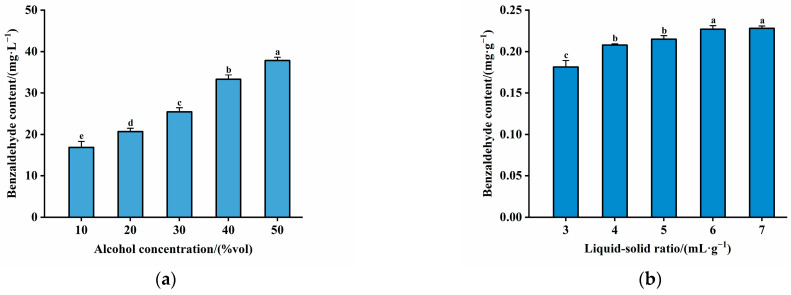
Effects of alcohol concentration and liquid–solid ratio on the extraction of benzaldehyde: (**a**) alcohol concentration; (**b**) liquid–solid ratio. Different letters indicate significant differences (*p* < 0.05).

The effects of alcohol concentration and solid-to-liquid ratio on the amount of benzaldehyde extracted from plum kernels were explored first. As shown in Figure 3a, it can be found that the alcohol concentration was positively correlated with benzaldehyde content. As the alcohol concentration increased, the benzaldehyde content increased significantly (*p* < 0.05). It is worth noting that the benzaldehyde content did not reach the leaching equilibrium when the alcohol concentration was 50% vol, and the existing law showed that the benzaldehyde content would continue to increase with further elevation of the alcohol concentration. However, due to the high concentration of alcohol, it masks the final plum wine aroma. The alcohol flavor is too prominent, and not conducive to controlling the degree of plum wine; thus, it is not suitable to continue to improve the alcohol concentration in reality. Therefore, 50% vol is preferred as the alcohol concentration condition for benzaldehyde extraction. Otherwise, the relationship between the food-grade alcohol liquid ratio and benzaldehyde extraction also indicated a direct positive correlation (Figure 3b). A notable increase in benzaldehyde content occurred with higher liquid ratios (*p* < 0.05), suggesting a proportional relationship between the liquid ratio and the extracted benzaldehyde quantity. Equilibrium in extraction was observed when the liquid–solid ratio surpassed 6. Consequently, a liquid–solid ratio (mL:g) of 6 was deemed optimal for subsequent benzaldehyde extraction experiments using plum kernels.

**Figure 4:**
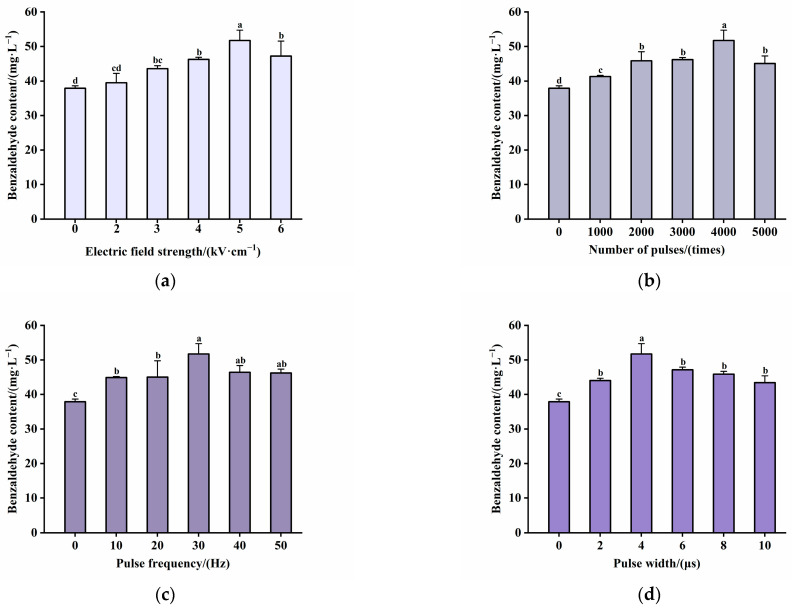
Effects of different parameters of PEF-assisted food-grade alcohol extraction on the benzaldehyde contents in the kernel of plums: (**a**) electric field strength; (**b**) number of pulses; (**c**) pulse frequency; (**d**) pulse width. Different letters indicate significant differences (*p* < 0.05).

Then, we explored the effects of different electric field strengths, number of pulses, pulse frequency, and width on the benzaldehyde extraction (Figure 4a–d). In the range of 0 to 5 kV/cm, a positive correlation was observed between electric field strength and benzaldehyde content. Notably, at 5 kV/cm, the experimental group showed a significant increase (*p* < 0.05) in benzaldehyde content, reaching 51.76 mg/L, a 36.75% rise compared to the control group (37.85 mg/L). However, at 6 kV/cm, benzaldehyde content decreased to 47.25 mg/L, possibly due to excessive field strength damaging the benzaldehyde (Figure 4a). Similarly, within the range of 0 to 4000 pulses, there was a positive correlation between the pulse number and benzaldehyde content. Increasing the pulses to 5000 led to a 12.85% reduction in benzaldehyde content compared to 4000 pulses (Figure 4b). Additionally, the experimental group with a pulse frequency of 30 Hz and a pulse width of 4 μs exhibited the highest benzaldehyde content in each group (*p* < 0.05) (Figure 4c,d).

In order to further optimize the parameters for PEF-assisted benzaldehyde extraction, the interactions of the four single factors were optimized using RSM (the complete experimental plan and the dependent variable values are provided in Tables S1 and S2). The second-order polynomial quadratic regression equation with benzaldehyde content as the indicator Y is as follows:

Y = 51.518 + 0.431A − 0.486B + 0.609C + 1.381D − 0.005AB + 0.008AC + 0.015AD − 0.010BC − 0.018BD + 0.020CD − 4.650A^2^ − 5.623B^2^ − 5.535C^2^ − 5.653D^2^ (1)

**Figure 5:**
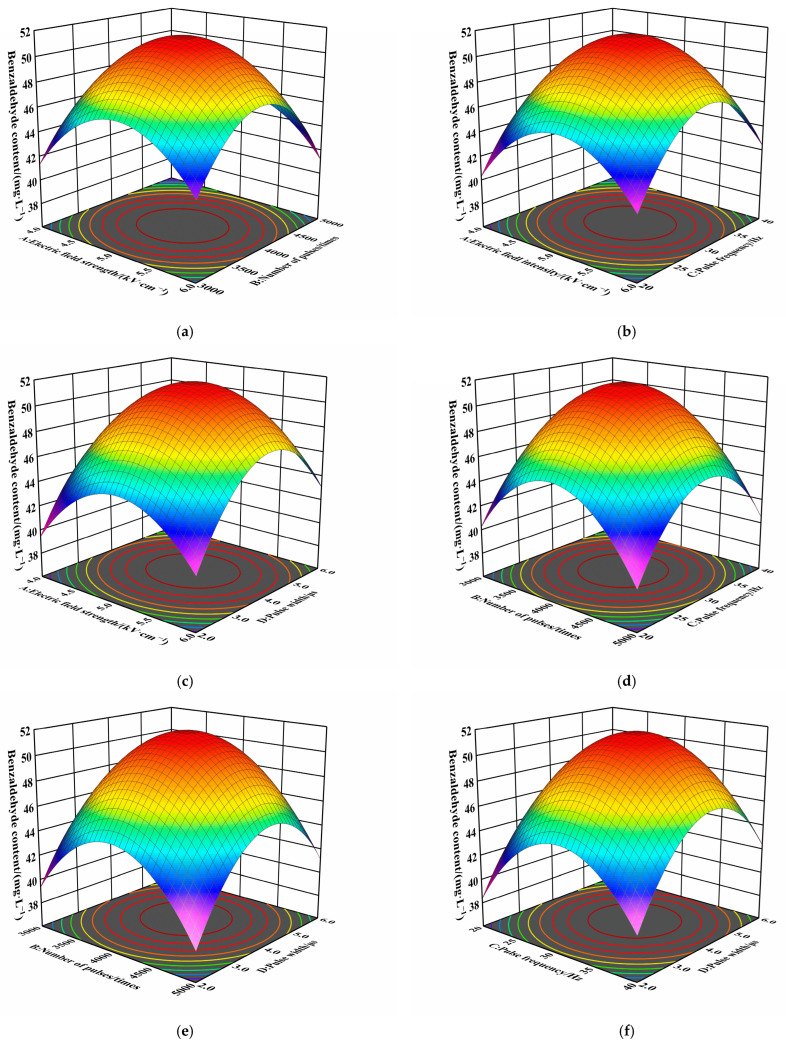
Response surface analysis of PEF-assisted benzaldehyde extraction: Interactive effects of (**a**) electric field strength and pulse number; (**b**) electric field strength and pulse frequency; (**c**) electric field strength and pulse width; (**d**) pulse number and pulse frequency; (**e**) pulse number and pulse width; and (**f**) pulse frequency and pulse width on benzaldehyde yield.

The experimental parameters and ANOVA results are shown in Table 4, which shows that the model has good accuracy, reliability, and experimental stability. In addition, the three-dimensional surface plot of the response surface model has obvious curved surfaces and corresponds to the previous one-factor trend, further indicating that the model is reliable (Figure 5a–f). According to the optimization results of the model and the actual equipment, 5 kV/cm electric field strength, 3957 pulses, 31 Hz pulse frequency, and 4 μs pulse width were selected as the optimized experimental conditions. The value of benzaldehyde content of 50.98 ± 0.16 mg/L was obtained under these conditions, which is basically the same as that predicted, and it can be used as a processing parameter for the subsequent experiments.

### 2.4. Changes in Benzaldehyde, Amygdalin, and Cyanide Concentrations During Kernel Soaking with PEF-Assisted Extraction

**Figure 6:**
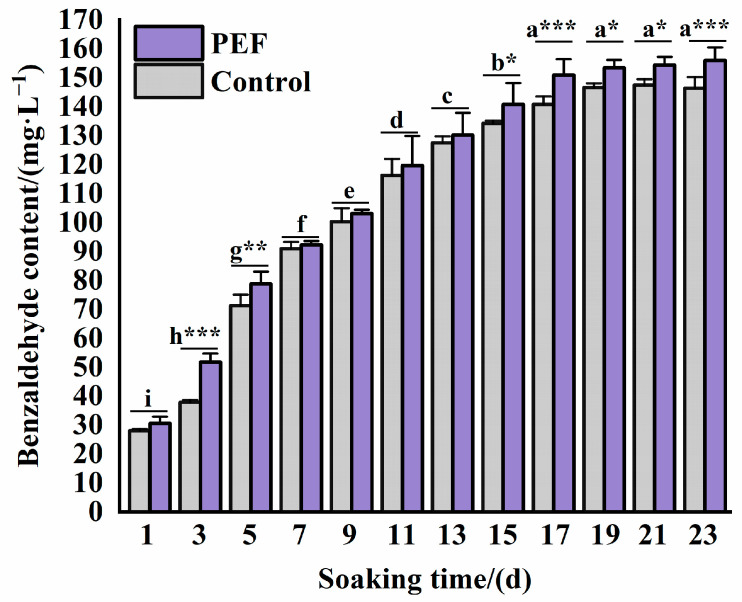
Comparison of changes in benzaldehyde content in plum kernel soaking solution after treatment with optimal PEF treatment parameters. Different letters indicate significant differences between different soaking times (*p* < 0.05). * indicates significant differences between PEF and fontrol for the same soaking time: * (*p* < 0.05), ** (*p* < 0.01), *** (*p* < 0.001).

The effectiveness of synergistic PEF treatment on benzaldehyde extraction from plum kernel was explored first. After a detoxification pretreatment, the plum kernels were soaked in 50% vol food-grade alcohol at a ratio of 6:1 (mL:g), in which the control group did not undergo the PEF treatment, and the PEF group was extracted with the optimized parameters (5 kV/cm, 31 Hz, 4 μs, 3957 pulses). As shown in Figure 6, the benzaldehyde contents in the soaking solution of the PEF group were greater than those of the control group for 1~23 days. At 17 days of immersion, the equilibrium contents of benzaldehyde in the soaking solution of the PEF group were 153.43 mg/L, while those of the control group were 146.64 mg/L at 19 days of immersion, indicating a 4.63% increase in benzaldehyde content in the PEF group compared to the control at equilibrium. The equilibrium time of benzaldehyde extraction can also be shortened by 10.53%. Lopez et al. [28] investigated the effects of PEF on the maceration time in elaboration of red wines, and they found that the total stilbenes, trans-resveratrol, and trans-piceid of Graciano wines obtained from PEF samples showed a higher concentration than the control wines, and significantly reduced the number of days of maceration required for red wines, noting that PEF is a technology available for use in wineries to create red wines with reduced maceration time. Moreover, the results of the study by Ntourtoglou et al. [29] showed that a PEF of 1.2 kV/cm increased the extraction efficiency of woody compounds from 5% to 200%, which resulted in wines with more mellow oak flavors, suggesting that PEF could be introduced as a new technology to accelerate the aging process in the wine, brandy, and whiskey industries.

**Figure 7:**
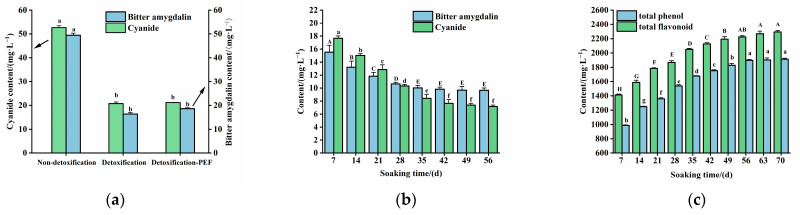
(**a**) Changes in the contents of harmful substances during the infusion of plum kernels after detoxification treatment; (**b**) changes in the contents of bitter amygdalin and cyanide in plum wine infused using the new process; (**c**) changes in the contents of total phenols and total flavonoids in plum wine infused using the new process. Different letters indicate significant differences (*p* < 0.05).

Since the PEF can continuously apply a pulsed high voltage to the cell wall and membrane within a short period of time to enhance their permeability, it can effectively improve the dissolution efficiency of intracellular components when treating plant materials, which was also verified in our benzaldehyde extraction experiments described above. Thus, based on this property of PEF, it is worthwhile to further investigate whether it also leads to some extent to an increase in the amount of amygdalin and cyanide dissolved in the subsequent soaking process, thus affecting the detoxification effect. Here, the detoxification of bitter amygdalin and cyanide during the 19-day alcohol immersion was evaluated. Figure 7a shows the contents of bitter amygdalin and cyanide in the soaking solution of plum kernels when equilibrium was reached, where the non-detoxification group indicates direct alcohol immersion without distilled water detoxification, the detoxification control group indicates alcohol immersion after detoxification, and the detoxification-PEF group indicates PEF synergistic alcohol infusion after detoxification. After detoxification pretreatment, the content of bitter amygdalin in the soaking solution of plum kernel was reduced from 49.42 mg/L to 16.42 mg/L, i.e., which in the detoxification-PEF group was 18.61 mg/L, increased by 2.19 mg/L compared to the detoxification group, but also reduced by 62.34% compared with the non-detoxification group. Meanwhile, the cyanide content in the soaking solution was 21.4 mg/L in the detoxification-PEF group, a 59.62% decrease compared to the non-detoxification group, and similar to the detoxification group (21.0 mg/L). Thus, the results indicate that PEF treatment also promotes the dissolution of harmful components to a certain extent, but due to PEF co-infiltration, it can significantly improve the extraction of benzaldehyde from the kernel and can effectively shorten the soaking cycle of plum wine. It was also 62.34% and 59.62% reduced in terms of bitter amygdalin and cyanide compared with the non-detoxification group, respectively, so PEF co-infiltration is effective and necessary.

### 2.5. Comparison of the Analysis of the Components of the New Process of Soaking Plum Wine

The above experimental results of detoxification showed that distilled water immersion could effectively remove bitter amygdalin and cyanide from plum kernels, and the optimal parameters of PEF-assisted extraction of benzaldehyde were determined by the response surface. Therefore, the new process of our soaking plum wine was determined: the whole plum kernels were first soaked in distilled water: kernel = 15:1 (mL:g) for 30 h at room temperature for detoxification pretreatment, and soaked in 50 vol% food-grade alcohol at a ratio of 6:1 (mL:g), then poured into the PEF treatment chamber with the electric field strength of 5 kV/cm, pulse frequency of 31 Hz, and pulse width of 4 μs for 3957 iterations. After PEF processing, the whole plum kernel alcohol-soaking solution was poured into a 5.0 L brown glass jar and soaked at room temperature for 17 days, then filtered through a filter cloth to obtain the plum kernel alcohol-soaking solution. A total of 1.5 kg of plum yellow flesh, 2.0 kg of sugar, and plum kernel alcohol-soaking solution were poured into a 10.0 L brown glass jar and soaked at room temperature to obtain plum wine.

#### 2.5.1. Evaluation of the Detoxification Effect of a New Process of Soaking Green Plum Wine

The changes in bitter amygdalin and cyanide contents during the soaking process of plum wine with the new process were examined (Figure 7b). It can be seen that the contents of bitter amygdalin and cyanide in the wine samples decreased continuously during the soaking process. After soaking for 7 to 35 days, the bitter amygdalin content decreased from 15.53 mg/L to 10.02 mg/L, and the cyanide content decreased from 17.7 mg/L to 8.4 mg/L. Considering that amygdalin and cyanide are mainly distributed in the kernels, the detoxification pretreatment of plum kernels removed most of the bitter amygdalin and cyanide (Figure 7a). During the flesh-soaking process, the water in the flesh continued to precipitate into the wine, continuously reducing the content of bitter amygdalin and cyanide in the wine. At the same time, the cyanide in the wine was volatile during the soaking process, which further reduced the cyanide content. The data showed that the equilibrium time of bitter amygdalin and cyanide content in the new process was 35 days and 42 days, respectively, and the equilibrium content was 10.02 mg/L and 8.4 mg/L. In summary, the new process of plum kernel detoxification has an obvious effect on reducing harmful components, and it is simple to operate, easy to implement, and has practical industrial value.

#### 2.5.2. Evaluation of Total Phenols and Total Flavonoids in a New Process of Soaking Green Plum Wine

Phenolics and flavonoids are secondary plant metabolites that play a key role in the organoleptic and nutritional quality of fruits, vegetables, and other plants [30], and these compounds and their antioxidant activity have long been associated with the beneficial effects of fruit wines [31]. The variational rules of total phenol and total flavonoid content of plum wine soaked using the new process are shown in Figure 7c; the total phenol and total flavonoid contents of the wine samples increased rapidly during the maceration process of the yellow plums, and on the 7th day of maceration, the total phenol and flavonoid contents reached 987.46 mg/L and 1412.70 mg/L, respectively. The total phenol content of the prune wine soaked using the new process increased from 987.46 to 1829.36 mg/L, and the total flavonoid content increased from 1412.70 to 2193.46 mg/L during the 7~49 days of maceration. The total phenol and total flavonoid contents reached leaching equilibrium after 56 days of maceration. In addition, the pretreatment of plum kernel maceration for water detoxification required 30 h, and the equilibrium time for extracting benzaldehyde from plum kernel was 17 days. Thus, the production cycle of the new process of soaking plum wine was about 75 days, which was 37.5% shorter than 120 days for the previous PEF—whole-fruit soaking plum wine [32]. In industrial production, if the kernel and plum steeping procedures are rationalized, the production cycle can be further shortened. Compared with whole-fruit-immersed plum wine, the new process has an obvious effect of shortening the production cycle and has practical industrial application value.

## 3. Discussion

In this work, a new process for the production of plum soaking wine was developed to reduce its content of bitter amygdalin and cyanide while increasing the solubility of the characteristic flavor substance, benzaldehyde, and further shortening the maceration production cycle. We first compared the differences in aroma components between plum kernels, green pulp, and yellow pulp, and the results showed that benzaldehyde was almost completely distributed in the kernels. In addition, the yellow pulp of plums had a total of 40 aroma components, a higher aroma activity value (OAV), and a pleasant floral and fruity aroma, which was superior to that of the green-skinned plums in all aspects. Therefore, we chose yellow pulp with a higher maturity as a macerating raw material. Subsequently, the plum kernel was separated from the yellow flesh and pretreated via water-soaked whole kernel detoxification. Then, the whole plum kernel was soaked in food-grade alcohol assisted by a pulsed electric field to obtain the characteristic aroma component, benzaldehyde, and the yellow flesh equilibrated by benzaldehyde soaking was soaked in a kernel alcohol-soaking solution to obtain the soaked plum wine.

The contents of bitter amygdalin and cyanide in plum kernels after detoxification were reduced by 62.34% and 59.62%, respectively, and further reduced during subsequent soaking using the new process. Moreover, the PEF synergistic extraction of benzaldehyde still showed an increase of 4.63% over the control group when the equilibration time was shortened by 10.53%. Otherwise, when the total phenol and total flavonoid contents reached equilibrium, the whole production cycle of the new process soaking plum wine showed a 37.5% shorter time than that of our previous PEF-whole fruit soaking plum wine cycle, which just needed about 75 days. PEF technology has shown considerable potential to improve the extraction efficiency of bioactive compounds in fruit wine production while reducing processing time. For example, Zheng et al. [33] demonstrated that PEF-assisted treatment significantly increased the dry extract content in fermented wine. In addition, a specific energy input of 22 kJ·kg^−1^ from PEF treatment promoted the release of grape aroma precursors, thereby enhancing the sensory profile of the wine [13]. In a study on “Cabernet Sauvignon” wine, applying a 5 kV·cm^−1^ electric field with a specific energy of 2.1 kJ·kg^−1^ improved color intensity, as well as the contents of anthocyanins, total phenolics, and tannins, regardless of maceration duration, while reducing the maceration time from 268 h to 72 h [34]. The study explored the solutions to the practical problems of the industrialized production of plum wine, which is of great significance for the practical industrial application of pulsed electric field and the production of plum wine.

## 4. Materials and Methods

### 4.1. Raw Materials and Chemicals

Green plums were provided by Luhe Guotai Green Plum Industry Development Co., Ltd. (Shanwei City, China). The medium-ripe plums were split into two groups: one portion was stored in cold storage at 4 °C, while the other was placed in a carton at room temperature until fully mature plums (yellow flesh) were obtained. Following thorough rinsing of both green and yellow flesh, an intelligent pitting machine with a cylindrical cutting tool in the pitting machine was employed to separate the plum kernel and flesh, ensuring the preservation of the integrity of the flesh. Consequently, the raw material of the plums was segmented into three distinct parts: the plum kernels, green flesh, and yellow flesh.

### 4.2. Determination of Aroma Components in the Kernel and Flesh of Plums

Following the approach outlined by Butkhup et al. [35], the determination of aroma components in the kernels, green flesh, and yellow flesh was conducted using headspace solid-phase microextraction (HS-SPME-GC-MS, the equipment was obtained from Agilent Technologies Inc., Santa Clara, CA, USA). After kernels, green flesh, and yellow flesh were broken with a medicine crusher. About 2.50 g of samples was weighed, combined with 1.00 g of NaCl, 0.50 g of CaCl_2_, and 60 μL of a 50.00 mg/L, 3-octanol internal standard solution (solvent: 3% ethanol) (all were purchased from Macklin Biochemical Co., Ltd., Shanghai, China). Subsequently, these mixtures were introduced into 20.0 mL threaded headspace vials to facilitate analysis.

The solid-phase microextraction process entailed the use of an extraction head (50/30 μm DVB/CAR/PDMS) that underwent aging at 260 °C for a duration of 60 min. Concurrently, the headspace vials were subjected to incubation at 45 °C for 20 min, followed by a 40 min extraction period at 45 °C utilizing the extraction head. After the extraction, the analysis was conducted at the GC inlet at 230 °C, employing a splitter ratio of 5:1 for a duration of 3 min. To prevent cross-contamination between samples, the extraction head underwent a 10 min aging process at 260 °C.

GC-MS analysis was carried out according to the method outlined by Wang et al. [32]. Firstly, the initial temperature was set at 40 °C and held for 5 min; then increased to 70 °C at 2 °C/min and held for 2 min; and then increased to 120 °C at 3 °C/min, 150 °C at 5 °C/min, and finally increased to 220 °C at 10 °C/min for 2 min with a transfer line temperature of 280 °C. The maminss detector was operated in EI mode with a voltage of 70 eV, an ion source of 230 °C, a scan rate of 2.88 scans/s, a mass detection range of m/z 29 to 540, and a carrier gas of helium at a flow rate of 2.25 mL/min. Finally, the aroma composition was determined by comparing with the relevant mass spectrometry data from the mass spectrometry database (NIST20) and related literature and quantified according to the relative content of the 3-octanol internal standard.

### 4.3. Detoxification Pretreatment of Plum Kernels

In order to thoroughly assess the impact of soaking duration and distilled water ratio on the removal of bitter amygdalin and cyanide from plum kernels, a detoxification rate was employed for comprehensive characterization. This rate, expressed as the ratio of removed harmful components to the original content, was calculated using Equation (2):

(2) $$Y=\frac{C_{0}-C_{1}}{C_{0}}$$

Here, Y denotes the detoxification rate, encompassing both cyanide and amygdalin detoxification rates. C_0_ and C_1_ represent the concentrations of these harmful components in the alcohol-soaking solution of green plum kernels after and before detoxification, respectively.

In the process of kernel detoxification pretreatment, whole plum kernels, ranging in mass from 15.00 to 16.00 g, were randomly selected and weighed. The samples were soaked in distilled water with a plum kernel ratio of 15:1 (mL:g) for durations of 0, 2, 4, 6, 8, 12, 18, 24, 30, and 36 h at room temperature. Then, the kernels were filtered with filter paper and further soaked in 50 vol% alcohol at a ratio of 1:10 (g:mL) for 12 h. The alcohol-soaking solution was then filtered to determine the concentrations of bitter amygdalin and cyanide. Subsequently, the detoxification rates of bitter amygdalin and cyanide were calculated using equation (1), optimizing the water-soaking time for the detoxification of plum kernels. Meanwhile, the kernels were soaked in distilled water: kernel = 5:1, 10:1, 15:1, 20:1, 25:1, and 30:1 (mL:g) for 30 h at room temperature, filtered through filter paper to obtain the kernel residue, and then soaked in kernel: 50 vol% alcohol = 1:10 (g:mL) for 12 h to obtain the alcoholic liquor soaking solution. The detoxification rate of amygdalin and cyanide was calculated, and the ratio of water immersion for the detoxification of plum kernel was preferred.

Here, the amygdalin levels were determined using high-performance liquid chromatography (HPLC) [36]. Then, 0.05 g of bitter amygdalin standard was accurately weighed, dissolved, and diluted with methanol to 50.00 mL, and a standard stock solution was prepared with a concentration of 1.00 mg/mL. A series of bitter amygdalin standard working solutions with concentrations of 500.00, 250.00, 100.00, 50.00, 25.00, 10.00, and 5.00 mg/L were prepared. The content of amygdalin in the wine samples was quantified using the external standard method, and the samples were diluted with methanol to the appropriate concentration so that the determined concentration fell within the range of the standard curve. The separation was performed on an Atlantis T3 column (4.6 mm × 250 mm, 5.0 μm) at a flow rate of 0.6 mL/min and a column temperature of 30 °C, with mobile phase A: 0.1% aqueous acetic acid and mobile phase B: acetonitrile. 60% to 5%; 17–20 min, 5% to 95%, maintained for 2 min; detection wavelength: 219 nm, injection volume: 5 μL; standard and sample solutions were filtered through a 0.22 μm microporous organic membrane before determination.

Meanwhile, cyanide content assessment was conducted based on the spectrophotometric method outlined in GB5009.36-2023 [37] Determination of Cyanide in Food. A total of 1.00 mL of sample was accurately absorbed in a 25 mL beaker, and 5 mL of NaOH solution was added (2 g/L), placed for 10 min, and then placed on a 160 °C~180 °C electric heating plate for low-temperature heating of the solution, leaving about 1 mL. It was rremoved and brought down to room temperature with 2 g/L of NaOH solution to transfer to a 10 mL stoppered cuvette, and finally, 2 g/L of NaOH solution was added to reach 5 mL. The prepared sample was measured using a 1 cm cuvette, with a 2 g/L NaOH solution as the blank solution to adjust the zero point at the wavelength of 638 nm to determine the absorbance.

### 4.4. PEF-Assisted Extraction of Benzaldehyde in Plum Kernels

First, the effects of food-grade alcohol concentration on benzaldehyde content were investigated. A total of 12 whole detoxified kernels were randomly weighed as a group, and their mass was recorded. The ratio of the extracted material and liquid in food-grade alcohol immersion was 6:1 (mL:g), and the benzaldehyde contents in the immersion solution were determined after 48 h of immersion in 10 vol%, 20 vol%, 30 vol%, 40 vol%, and 50 vol% of food-grade alcohol, respectively, so as to optimize the concentration of the immersed food-grade alcohol. Afterward, 50 vol% food-grade alcohol was used for soaking, and the material–liquid ratios were 3:1, 4:1, 5:1, 6:1, and 7:1 (mL:g), respectively. The benzaldehyde content in the soaking solution was determined after 48 h so as to optimize the ratio of the extracted food-grade alcohol.

Next, the characteristic aroma component, benzaldehyde, from plum kernels was extracted using PEF-assisted alcohol soaking. The study delved into the effects of four individual factors—electric field strength, pulse number, pulse frequency, and pulse width—on the benzaldehyde content in the soaking solution. To quantify the benzaldehyde content in the soaking solution, the determination method described by Gao et al. was used [38]. This method involved utilizing high-performance liquid chromatography (HPLC) to assess benzaldehyde levels in plum wine samples, ensuring accurate quantification of benzaldehyde in the wine samples derived from the soaking process.

To investigate the effects of electric field strength on benzaldehyde content, the detoxified kernels were subjected to PEF treatment at a pulse frequency of 30 Hz and a pulse width of 4 μs; with electric field strengths of 2, 3, 4, 5, and 6 kV/cm, respectively; and pulsed 4000 times. Then, the effects were studied for the number of pulses, with kernels treated with PEF at an electric field strength of 5 kV/cm, a pulse frequency of 30 Hz, and a pulse width of 4 μs, using 1000, 2000, 3000, 4000, and 5000 pulses, respectively. Kernels used to explore the effects of pulse frequency on benzaldehyde content were examined under PEF conditions of electric field strength of 5 kV/cm; pulse width of 4 μs; and pulse frequency of 10, 20, 30, 40, and 50 Hz, respectively, with 4000 pulses. Finally, the effect of pulse width on benzaldehyde content was investigated by treating the kernels at an electric field strength of 5 kV/cm; a pulse frequency of 30 Hz; pulse widths of 2, 4, 6, 8, and 10 μs; and 4000 pulses. After the pulse treatment, the alcohol-soaking solution of the kernel of green plum was poured back and then soaked at room temperature for 48 h to determine the benzaldehyde content. In order to investigate the interaction between each single factor—electric field strength (A), number of pulses (B), pulse frequency (C), and pulse width (D)—to screen the optimal treatment conditions, a mathematical model was developed using response surface analysis (RSM). In this study, a Box–Behnken test was used for the response surface experimental design and analyzed using Design-Expert software (version 13).

### 4.5. Effect of PEF-Assisted Extraction on the Content of Benzaldehyde, Amygdalin, and Cyanide During Kernel Soaking

The results of previous studies, as well as our experimental data, showed that PEF can effectively dissolve components in the material and be used to investigate the effect of PEF-assisted extraction on pre-detoxification and assess the efficacy of PEF-assisted extraction of benzaldehyde. Three experimental groups (non-detoxification group, detoxification group, and detoxification-PEF group) were used. In the non-detoxification group, 25 whole kernels were randomly weighed, then soaked in 50 vol% food alcohol at a ratio of 6:1 (mL:g). The detoxification group involved soaking 25 plum kernels in a 15:1 (mL:g) ratio of distilled water for 30 h; followed by filtration; then soaked in 50 vol% food-grade alcohol at a 6:1 (mL:g) ratio at room temperature, in which the samples were taken once on each of the 1, 3, 5, 7, 9, 11, 13, 15, 17, 19, 21, and 23 days of soaking to determine the benzaldehyde content. Similarly, in the detoxification-PEF group, plum kernels were detoxified in distilled water and then soaked in 50 vol% alcohol at a ratio of 6:1 (mL:g) and then poured into the PEF treatment chamber and processed under the optimized parameters. Then, the alcohol-soaking solution was poured back and soaked at room temperature, and the samples were taken once on each of the 1, 3, 5, 7, 9, 11, 13, 15, 17, 19, 21, and 23 days of soaking to determine the benzaldehyde content. Moreover, all three controls were assayed for amygdalin and cyanide after 19 days of immersion.

### 4.6. Preparation of Soaked Plum Wine with New Processing Techniques

After determining the optimal parameters for developing a new process for soaking plum wine, production of the new process plum wine was started. Throughout the maceration process, we further followed up with the determination of the content of bitter amygdalin and cyanide, as well as the content of total phenolics and total flavonoids in the body of the wine. Determination of bitter amygdalin and cyanide content began at 7 days of maceration, after which the wines were taken at 7-day intervals for determination, as was the case for total phenols and total flavonoids.

The content of total phenolic compounds in the wine was determined using the Folin–Ciocalteu (FC) method. First, 100 μL of sample or standard (gallic acid (Aladdin Biochemical Technology Co., Ltd., Shanghai, China)) was mixed with 1750 μL of distilled water, 200 μL of Folin–Ciocalteu reagent (Macklin Biochemical Co., Ltd., Shanghai, China) (dilution ratio 1:10, *v*/*v*), and 1000 μL of 15% Na_2_CO_3_ solution. Then, it was incubated for 2 h at room temperature in the dark, and the absorbance was measured at 765 nm [39]. The content of total flavonoids was determined by referring to the method outlined in [40]; specifically, 1.0 mL of sample or standard (quercetin) was mixed with 4.0 mL of water and 3.0 mL of 5% NaNO_2_ solution, and after 5 min, 0.3 mL of 10% AlCl_3_ was added. Then, the sample was mixed, and after 6 min, neutralized with 2.0 mL of 1 M NaOH solution. It stood at room temperature for 10 min, and the absorbance was measured at 510 nm using a UV-2450 spectrophotometer (Shimadzu, Tokyo, Japan).

### 4.7. Statistical Analyses

Each treatment process was performed in triplicate, and the result was presented as the mean ± standard deviation (SD). Data were analyzed by variance (One-way/Two-way ANOVA) followed by a Tukey test using SPSS 22.0 (IBM, Armonk, NY, USA), and *p* < 0.05 was considered significantly different.

## Figures and Tables

**Table 1:** Determination of aroma components in kernel of green plum.

Peak	Name	CAS	RT	Aroma Description	Relative Content (mg·kg^−1^)	Odor Detection Threshold (mg·kg^−1^) [18]	OAV
1	Benzaldehyde	100-52-7	31.838	The fragrances of bitter almonds, cherries, and nuts	2024.66	0.35	5784.75
2	Benzyl alcohol	100-51-6	45.071	Faint honey-sweet fruit aroma	35.89	10.00	3.59

RT: retention time; CAS: Chemical Abstracts Service; OAV: Odor Activity Value (OAV).

**Table 2:** Determination of aroma components in green flesh of plum.

Peak	Name	CAS	RT	Aroma Description	Relative Content (mg/kg^−1^)	Odor Detection Threshold (mg/kg^−1^)	OAV
1	Propionic ether	105-37-3	4.038	Pineapple fragrance	0.0028	0.01	0.2800
2	Valeraldehyde	110-62-3	4.481	—	0.0213	0.042	0.5071
3	Ethyl butyrate	105-54-4	6.063	Pineapple fragrance	0.1062	0.001	106.20
4	4-hexene-3-ketone	2497-21-4	7.073	—	0.0114	—	—
5	Butyl acetate	123-86-4	7.373	Delightful pineapple and banana fragrance	0.0112	0.066	0.1697
6	Hexanal	66-25-1	7.692	The smell of fresh green grass	0.0281	0.0045	6.2444
7	Limetol	7392-19-0	8.699	The refreshing aroma of camphor, sandalwood, and white lemon	0.0662	—	—
8	Terpinene	99-86-5	10.416	The citrusy, lemon-like fragrance of oranges	0.0346	0.08	0.4325
9	Myrcene	123-35-3	11.486	A light resinous fragrance	0.0047	0.015	0.3133
10	D-limonene	5989-27-5	12.945	The light fragrance of fresh flowers	0.2471	0.2	1.2355
11	Ethyl hexanoate	123-66-0	15.446	The aroma of pineapple and banana fruits	0.0261	0.27	0.0967
12	Ocimene quintoxide	7416-35-5	15.754	The fresh and cool taste of citrus, lime flavor	0.0124	—	—
13	m-Cymene	99-87-6	17.102	The strong smell of carrots	0.0024	0.4	0.0060
14	Ethyl 5-Hexenoate	1000302-89-9	18.098	—	0.0016	—	—
15	3-oxo-1-heptene	2918-13-0	19.361	—	0.0499	0.007	7.1286
16	Heptenal	18829-55-5	20.569	A green grass fragrance	0.1399	0.013	10.7615
17	Methylheptenone	110-93-0	21.667	A fresh fruits fragrance	0.0365	0.05	0.7300
18	(2E)-2-Octenal	2548-87-0	27.308	A cucumber fragrance	0.0547	0.003	18.2333
19	Ionene	475-03-6	27.698	—	0.0138	—	—
20	Ethyl caprylate	106-32-1	27.962	The fragrance of brandy	0.0069	0.2	0.0345
21	Linalool oxide	34995-77-2	28.108	The fragrances of sandalwood, floral scents, and camphor	0.0099	0.06	0.1650
22	Oct-1-en-3-ol	3391-86-4	29.038	The fragrances of lavender, rose, and hay	0.0353	0.001	35.3000
23	5-Methylnonan-5-ol	33933-78-7	29.577	—	0.0136	—	—
24	2,4-Heptadienal	4313-03-5	30.763	—	0.0312	10	0.0031
25	Benzaldehyde	100-52-7	31.905	The fragrances of bitter almonds, cherries, and nuts	0.0729	0.35	0.2083
26	Linalool	78-70-6	33.864	The fragrance of lily of the valley	0.0448	1.082	0.0414
27	Dihydrolinalool	29957-43-5	36.569	Rosewood oil fragrance	0.0298	—	—
28	Cis-β-Ocimene	7643-59-6	38.468	—	0.0251	—	—
29	2-Methylbutyric acid	116-53-0	38.781	Cheesy and fruity fragrance	0.0069	5.8	0.0012
30	Trans-terpin	7643-60-9	39.373	—	0.0312	—	—
31	α-Terpineol	98-55-5	39.811	The fragrance of cloves	0.0333	0.33	0.1009
32	β-Damascenone	23726-93-4	43.531	An intense rose fragrance	0.0046	0.002	2.3000
33	γ-Decalactone	706-14-9	49.587	A delightful fruity fragrance	0.0076	0.088	0.0864
34	Olivetol	500-66-3	49.678	—	0.0050	—	—
35	3,5-Di-tert-butylphenol	1138-52-9	51.387	—	0.0045	—	—
36	Benzoic acid	65-85-0	52.573	Faint bitter almond fragrance	0.0190	1.0	0.0190

“—” indicates that no data or statistics are available; RT: retention time; CAS: Chemical Abstracts Service; OAV: Odor Activity Value (OAV).

**Table 3:** Determination of aroma components in yellow flesh of green plum.

Peak	Name	CAS	RT	Aroma Description	Relative Content (mg·kg^−1^)	Odor Detection Threshold (mg·kg^−1^)	OAV
1	Ethyl butyrate	105-54-4	6.036	A pineapple fragrance	0.3470	0.001	347.0000
2	Butyl acetate	123-86-4	7.396	A delightful pineapple and banana fragrance	2.0667	0.066	31.3136
3	Limetol	7392-19-0	8.677	The refreshing aroma of camphor, sandalwood, and white lemon	0.0769	—	—
4	Amyl butyrate	540-18-1	9.467	An apricot fragrance	0.0102	0.21	0.0486
5	α-Terpinene	99-86-5	10.376	A citrusy, lemon fragrance	0.0623	0.08	0.7788
6	Pentyl acetate	628-63-7	12.053	A banana fragrance	0.0249	8.2	0.0030
7	Butyl butanoate	109-21-7	14.496	A pineapple fragrance	0.3704	1.089	0.3401
8	Ethyl hexanoate	123-66-0	15.41	The fruity aroma of pineapple and banana	0.1893	0.27	0.7011
9	Ocimene quintoxide	7416-35-5	15.711	The fresh and cool taste of citrus, lime flavor	0.0263	—	—
10	Hexyl acetate	142-92-7	17.776	The aroma of pear and apple	0.6151	0.002	307.5500
11	Ethyl 5-Hexenoate	1000302-89-9	18.06	—	0.0458	—	—
12	Ethyl hex-3-enoate	2396-83-0	19.492	The fragrance of pineapple	0.0070	—	—
13	(3Z)-3-Hexen-1-yl acetate	3681-71-8	20.395	A strong grassy fragrance	0.4138	0.031	13.3484
14	5-Hexenyl Acetate	5048-26-0	21.042	—	0.0434	—	—
15	Hex-2-enyl acetate	2497-18-9	21.577	The fragrance of fresh grass	0.0478	—	—
16	3-methylpentanol	589-35-5	23.064	—	0.0420	1	0.0420
17	Butyl Hexanoate	626-82-4	26.644	A pineapple fragrance	0.0920	0.7	0.1314
18	Hexyl butyrate	2639-63-6	26.817	A fruity fragrance	0.0819	0.25	0.3276
19	Ionene	475-03-6	27.663	—	0.0444	—	—
20	Ethyl caprylate	106-32-1	27.911	The aroma of brandy	0.0335	0.2	0.1675
21	5-methyl-5-nonanol	33933-78-7	29.536	—	0.0197	—	—
22	Benzaldehyde	100-52-7	31.846	The fragrances of bitter almonds, cherries, and nuts	0.2074	0.35	0.5926
23	Linalool	78-70-6	33.815	The fragrances of lily of the valley	0.1013	1.082	0.0936
24	Hexyl hexanoate	6378-65-0	36.379	The fragrances of green bean and raw fruit aromas	0.0098	0.5	0.0196
25	Hexyl octanoate	1551-42-4	36.517	—	0.0540	—	—
26	(Z)-β-ocimene	7643-59-6	38.42	—	0.0490	—	—
27	2-Methylbutyric acid	116-53-0	38.72	Cheesy and fruity flavors	0.0240	5.8	0.0041
28	(E)-β-ocimene	7643-60-9	39.334	—	0.0598	—	—
29	γ- Caprolactone	695-06-7	39.549	The fragrances of sweet herb with caramel aroma	0.0413	1.6	0.0258
30	α-Terpineol	98-55-5	39.769	The fragrance of cloves	0.0650	0.33	0.1970
31	Benzyl acetate	140-11-4	40.75	The fragrance of jasmine	0.0348	1.0	0.0348
32	Methyl salicylate	119-36-8	42.08	The fragrance of holly leaf	0.0056	0.04	0.1400
33	β-Damascenone	23726-93-4	43.504	An intense rose fragrance	0.0090	0.002	4.5000
34	Benzyl alcohol	100-51-6	45.071	A faint sweet fruit aroma	0.0141	10.0	0.0014
35	Octanoic acid	124-07-2	48.387	A fruity aroma	0.0107	3.0	0.0036
36	γ-Decalactone	706-14-9	49.571	A delightful fruity fragrance	0.1387	0.088	1.5761
37	Olivetol	500-66-3	49.667	—	0.0086	—	—
38	δ-Decalactone	705-86-2	50.182	The fragrances of cream, nut, sweet fruit	0.0060	0.16	0.0375
39	γ- Dodecalactone	2305-05-7	52.138	The fragrances of intense peach fruit, slight cream	0.0090	0.007	1.2857
40	Benzoic acid	65-85-0	52.554	Faint bitter almond fragrance	0.0103	1.0	0.0103

**Table 4:** Box–Behnken (BBD) design of four variables and response values.

Variant	−1	0	1
Electric field strength (A)	4 kV·cm^−1^	5 kV·cm^−1^	6 kV·cm^−1^
Number of pulses (B)	3000 times	4000 times	5000 times
Pulse frequency (C)	20 Hz	30 Hz	40 Hz
Pulse width (D)	2 μs	4 μs	6 μs
Model significance	<0.01	significance	
R^2^	0.9979	R^2^ Predicted	0.9964
R^2^ adjusted	0.9982	F-value	2606.41
C.V%	1.28		

## Data Availability

Data are contained within the article and Supplementary Materials.

## Supplementary Materials

The following supporting information can be downloaded at: https://www.mdpi.com/article/10.3390/molecules30224393/s1, Table S1. Box-Behnken (BBD) design of four variables and response values. Table S2. Box-Behnken (BBD) design of four variables and the dependent variable values corresponding to each point.

## Abbreviations

The following abbreviations are used in this manuscript:

PEF	Pulsed Electric Field.
OAV	Odor Activity Value.
