# Supplementary material for: Enhancing Plum Wine Safety and Aroma Using Pulsed Electric Field Pretreatment
Source: Molecules. 2025 Nov 13;30(22):4393. doi: 10.3390/molecules30224393 (PMC12654766; doi:10.3390/molecules30224393)
Supplement: Supplementary file 1 [file molecules-30-04393-s001.zip › molecules-3932882-supplementary.pdf]

Table S1. Box-Behnken (BBD) design of four variables and response values

Name	Units	Type	Changes	Std. Dev.	Low	High
Electric field strength	kV/cm	Factor	Easy	0	4	6
Number of pulses	times	Factor	Easy	0	3000	5000
Frequency	Hz	Factor	Easy	0	20	40
Pulse width	μs	Factor	Easy	0	2	6
Benzaldehyde content	mg/L	Response			38.37	51.76

Table S2. Box-Behnken (BBD) design of four variables and the dependent variable values corresponding to each point

std	Run	Factor 1<br>A:Electric field strength<br>(kV/cm)	Factor 2<br>B:Number of pulses<br>(times)	Factor 3<br>C:Frequency<br>(Hz)	Factor 4<br>D:Pulse width<br>(μs)	Response 1<br>Benzaldehyde content<br>(mg/L)
1	7	4	3000	30	4	41.30
2	9	6	3000	30	4	42.17
3	15	4	5000	30	4	40.33
4	27	6	5000	30	4	41.18
5	18	5	4000	20	2	38.37
6	25	5	4000	40	2	39.54
7	29	5	4000	20	6	41.08
8	19	5	4000	40	6	42.33
9	10	4	4000	30	2	39.40
10	2	6	4000	30	2	40.23
11	16	4	4000	30	6	42.17
12	13	6	4000	30	6	43.06
13	6	5	3000	20	4	40.23
14	3	5	5000	20	4	39.28
15	28	5	3000	40	4	41.46
16	8	5	5000	40	4	40.47
17	12	4	4000	20	4	40.29
18	23	6	4000	20	4	41.14
19	20	4	4000	40	4	41.51
20	5	6	4000	40	4	42.39
21	24	5	3000	30	2	39.34
22	22	5	5000	30	2	38.41
23	11	5	3000	30	6	42.11
24	17	5	5000	30	6	41.11
25	21	5	4000	30	4	51.68
26	14	5	4000	30	4	51.36
27	1	5	4000	30	4	51.76
28	4	5	4000	30	4	51.56
29	26	5	4000	30	4	51.23
